# Crystal structure, Hirshfeld surface analysis and inter­action energy and DFT studies of 2-chloro­ethyl 2-oxo-1-(prop-2-yn-1-yl)-1,2-di­hydro­quinoline-4-carboxyl­ate

**DOI:** 10.1107/S2056989019012283

**Published:** 2019-09-06

**Authors:** Sonia Hayani, Yassir Filali Baba, Tuncer Hökelek, Fouad Ouazzani Chahdi, Joel T. Mague, Nada Kheira Sebbar, Youssef Kandri Rodi

**Affiliations:** a Laboratoire de Chimie Organique Appliquée, Université Sidi Mohamed Ben Abdallah, Faculté des Sciences et Techniques, Route d’Immouzzer, BP 2202, Fez, Morocco; b Department of Physics, Hacettepe University, 06800 Beytepe, Ankara, Turkey; c Department of Chemistry, Tulane University, New Orleans, LA 70118, USA; d Laboratoire de Chimie Bioorganique Appliquée, Faculté des Sciences, Université Ibn Zohr, Agadir, Morocco

**Keywords:** crystal structure, quinoline, alkyne, hydrogen bond, π-stacking, Hirshfeld surface

## Abstract

The title compound, C_15_H_12_ClNO_3_, consists of a 1,2-di­hydro­quinoline-4-carb­oxyl­ate unit with 2-chloro­ethyl and propynyl substituents, where the quinoline moiety is almost planar and the propynyl substituent is nearly perpendicular to its mean plane. In the crystal, the mol­ecules form zigzag stacks along the *a*-axis direction through slightly offset π-stacking inter­actions between inversion-related quinoline moieties which are tied together by inter­molecular C—H_Prpn­yl_⋯O_Carbx_ and C—H_Chlethy_⋯O_Carbx_ (Prpnyl = propynyl, Carbx = carboxyl­ate and Chlethy = chloro­eth­yl) hydrogen bonds. The Hirshfeld surface analysis of the crystal structure indicates that the most important contributions for the crystal packing are from H⋯H (29.9%), H⋯O/O⋯H (21.4%), H⋯C/C⋯ H (19.4%), H⋯Cl/Cl⋯H (16.3%) and C⋯C (8.6%) inter­actions. Hydrogen bonding and van der Waals inter­actions are the dominant inter­actions in the crystal packing. Computational chemistry indicates that in the crystal, the C—H_Prpn­yl_⋯O_Carbx_ and C—H_Chlethy_⋯O_Carbx_ hydrogen bond energies are 67.1 and 61.7 kJ mol^−1^, respectively. Density functional theory (DFT) optimized structures at the B3LYP/ 6–311 G(d,p) level are compared with the experimentally determined mol­ecular structure in the solid state. The HOMO–LUMO behaviour was elucidated to determine the energy gap.

## Abstract 2

The title compound consists of a 1,2-di­hydro­quinoline-4-carboxyl­ate unit with 2-chloro­ethyl and propynyl substituents, where the quinoline moiety is almost planar and the propynyl substituent is nearly perpendicular to its mean plane. In the crystal, the mol­ecules form zigzag stacks along the *a*-axis direction through slightly offset π-stacking inter­actions between inversion-related quinoline moieties, which are tied together by inter­molecular C—H_Prpn­yl_⋯O_Carbx_ and C—H_Chlethy_⋯O_Carbx_ (Prpnyl = propynyl, Carbx = carboxyl­ate and Chlethy = chloro­eth­yl) hydrogen bonds.

## Chemical context   

The quinoline ring system is an important structural unit in naturally occurring quinoline alkaloids, therapeutics and synthetic analogues with inter­esting biological activities. Quinolone derivatives possess a variety of pharmacological properties such as anti-bacterial (Hu *et al.*, 2017*a* ▸; Zhang *et al.*, 2018 ▸), anti-tubercular (Fan *et al.*, 2018*a* ▸; Xu *et al.*, 2017 ▸), anti-malarial (Fan *et al.*, 2018*b* ▸; Hu *et al.*, 2017*b* ▸), anti-HIV (Sekgota *et al.*, 2017 ▸; Luo *et al.*, 2010 ▸), anti-HCV (Mandroni *et al.*, 2014 ▸; Cheng *et al.*, 2016 ▸) and anti-cancer (Pommier *et al.*, 2010 ▸; Shahin *et al.*, 2018 ▸; Bisacchi & Hale, 2016 ▸) activities. Recently, substituted quinolines have also been reported to act as antagonists for endothelin (Cheng *et al.*, 1996 ▸), 5HT3 (Anzini *et al.*, 1995 ▸), NK-3 (Giardina *et al.*, 1997 ▸) and leukotriene D4 (Gauthier *et al.*, 1990 ▸) receptors. They are also used as inhibitors of gastric (H^+^/K^+^)-ATPase (Ife *et al.*, 1992 ▸), di­hydro­orotate de­hydrogenase (Chen *et al.*, 1990 ▸) and 5-lipoxygenase (Musser *et al.*, 1987 ▸). As a continuation of our research on the development of *N*-substituted quinoline derivatives and the assessments of their potential pharmacological activities (Filali Baba *et al.*, 2016 ▸, 2017 ▸, 2019 ▸; Bouzian *et al.*, 2018 ▸, 2019*a* ▸), we have studied the condensation reaction of propargyl bromide with 2-chloro­ethyl 2-oxo-1,2-di­hydro­quinoline-4-carboxyl­ate under phase-transfer catalysis conditions using tetra-*n*-butyl­ammonium bromide (TBAB) as catalyst and potassium carbonate as base. We report herein on the synthesis and the mol­ecular and crystal structures of the title compound along with the Hirshfeld surface analysis and the inter­molecular inter­action energies and the density functional theory (DFT) computational calculation carried out at the B3LYP/6–311 G(d,p) level.
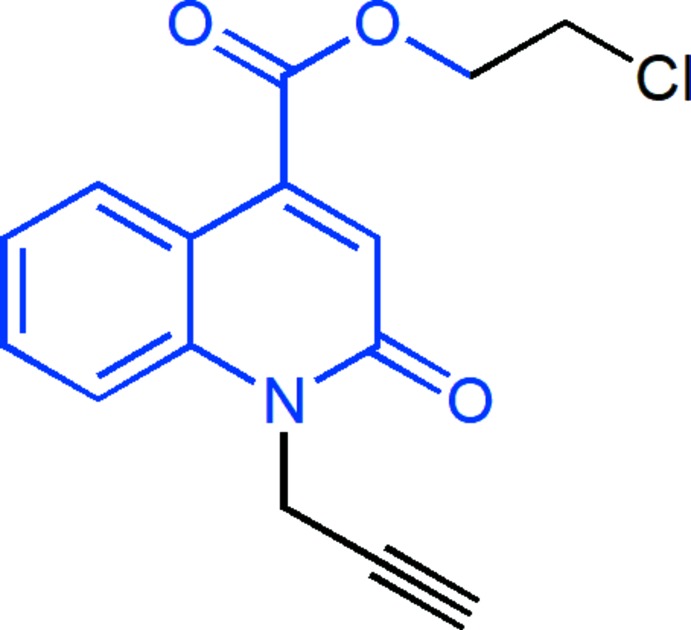


## Structural commentary   

**Figure 1:**
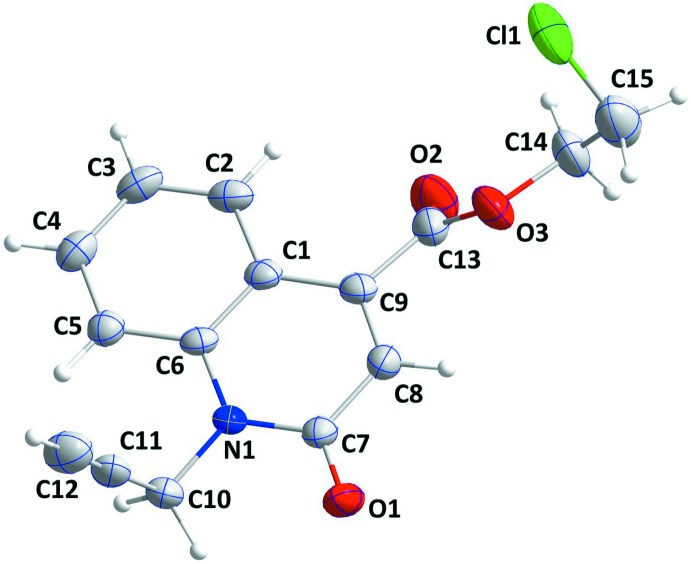
The mol­ecular structure of the title compound with the atom-numbering scheme. Displacement ellipsoids are drawn at the 50% probability level.

The title mol­ecule consists of a 1,2-di­hydro­quinoline-4-carboxyl­ate unit with 2-chloro­ethyl and propynyl substituents (Fig. 1 ▸). The constituent rings, *A* (C1–C6) and *B* (N1/C1/C6–C9), of the di­hydro­quinoline unit are oriented at a dihedral angle of 2.69 (17)°. The mean plane through the di­hydro­quinoline unit is almost planar with a maximum deviation of 0.040 (3) Å for atom N1, and the propynyl substituent is nearly perpendicular to that plane, the C6—N1—C10—C11 torsion angle being −79.6 (4)°. The carboxyl group is twisted out of coplanarity with the di­hydro­quinoline unit by a dihedral angle of 47.13 (23)°; this is also indicated by the C1—C9—C13—O2 torsion angle of −44.2 (6)°.

## Supra­molecular features   

**Figure 2:**
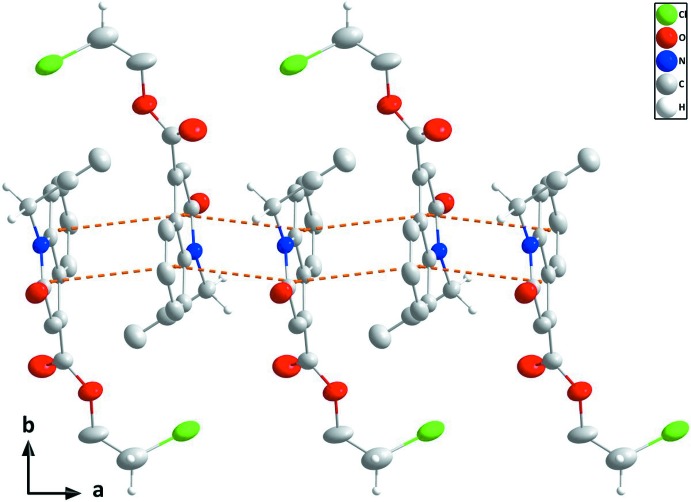
A partial packing diagram viewed along the *c*-axis direction with the π-stacking inter­actions shown as dashed lines.

**Figure 3:**
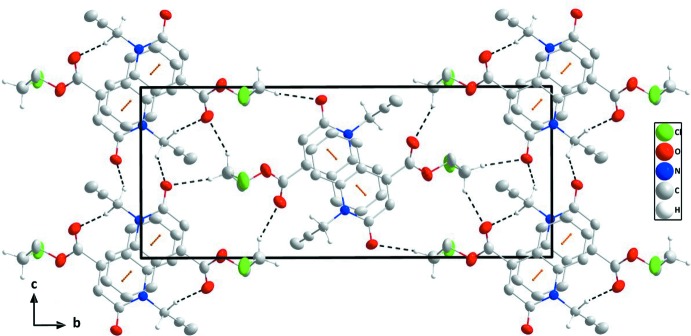
A partial packing diagram viewed along the *a*-axis direction with the C—H_Prpn­yl_⋯O_Carbx_ and C—H_Chlethy_⋯O_Carbx_ (Prpnyl = propynyl, Carbx = carboxyl­ate and Chlethy = chloro­eth­yl) hydrogen bonds and π-stacking inter­actions shown, respectively, as black and orange dashed lines.

In the crystal, the mol­ecules form zigzag stacks along the *a*-axis direction through slightly offset π-stacking inter­actions between inversion-related quinoline moieties (Fig. 2 ▸). The stacks are tied together by a network of inter­molecular C—H_Prpn­yl_⋯O_Carbx_ and C—H_Chlethy_⋯O_Carbx_ (Prpnyl = propynyl, Carbx = carboxyl­ate and Chlethy = chloro­eth­yl) hydrogen bonds, enclosing 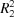
(16) and 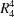
(8) ring motifs (Table 1 ▸ and Fig. 3 ▸). The π–π contacts between the constituent rings, *A* (C1–C6) and *B* (N1/C1/C6–C9), of the di­hydro­quinoline unit, *Cg2*⋯*Cg*1^i^, *Cg*2⋯*Cg*1^ii^ and *Cg*1⋯*Cg*1^i^ [centroid–centroid distance = 3.728 (2), 3.571 (2) and 3.761 (2) Å, respectively, where *Cg*1 and *Cg*2 are the centroids of the rings, *A* and *B*; symmetry codes: (i) 1 − *x*, 1 − *y*, 1 − *z* and (ii) −*x*, 1 − *y*, 1 − *z*], may further stabilize the structure.

## Hirshfeld surface analysis   

**Figure 4:**
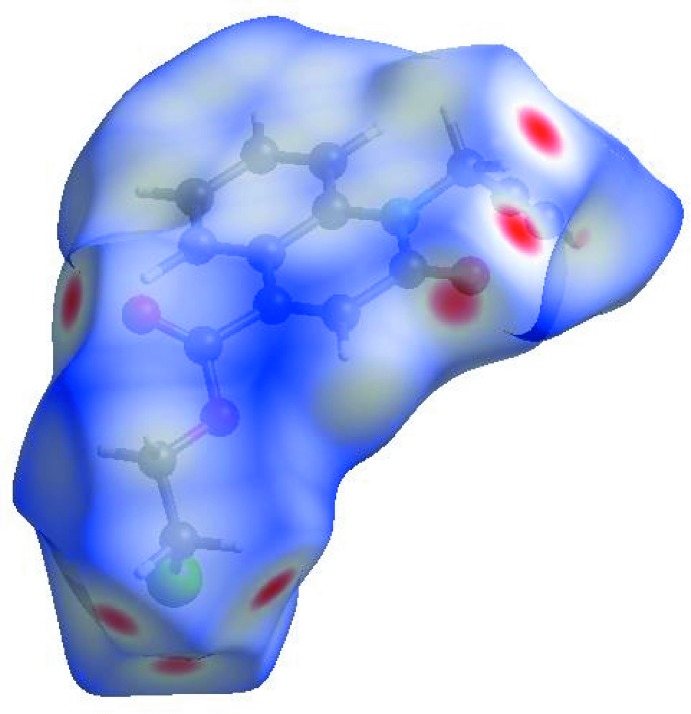
View of the three-dimensional Hirshfeld surface of the title compound plotted over *d*_norm_ in the range −0.2177 to 1.3626 a.u.

**Figure 5:**
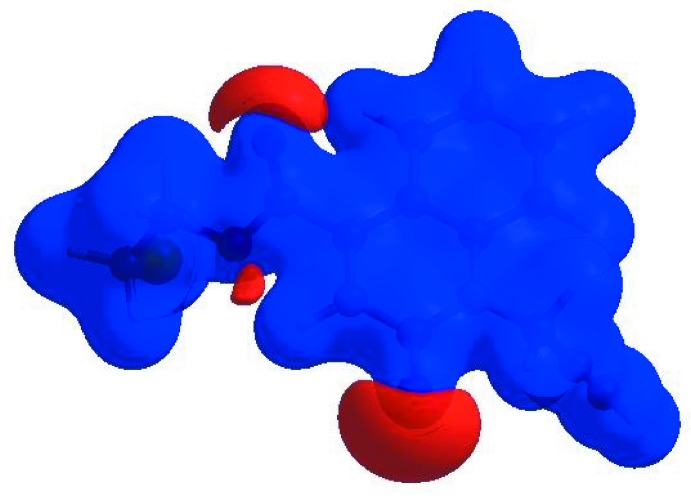
View of the three-dimensional Hirshfeld surface of the title compound plotted over electrostatic potential energy in the range −0.0500 to 0.0500 a.u. using the STO-3 G basis set at the Hartree–Fock level of theory. Hydrogen-bond donors and acceptors are shown as blue and red regions around the atoms, corresponding to positive and negative potentials, respectively.

**Figure 6:**
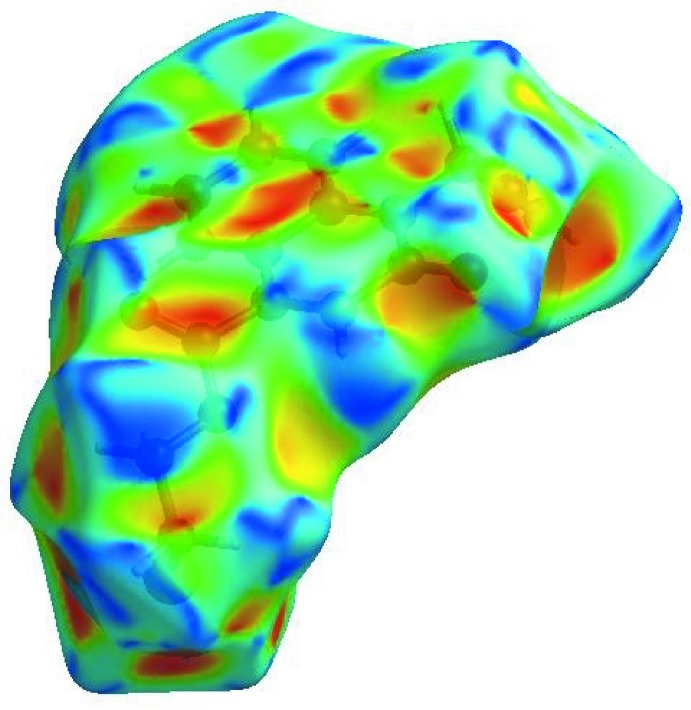
Hirshfeld surface of the title compound plotted over shape-index.

In order to visualize the inter­molecular inter­actions in the crystal of the title compound, a Hirshfeld surface (HS) analysis (Hirshfeld, 1977 ▸; Spackman & Jayatilaka, 2009 ▸) was carried out by using *CrystalExplorer17.5* (Turner *et al.*, 2017 ▸). In the HS plotted over *d*_norm_ (Fig. 4 ▸), the white surface indicates contacts with distances equal to the sum of van der Waals radii, and the red and blue colours indicate distances shorter (in close contact) or longer (distinct contact) than the van der Waals radii, respectively (Venkatesan *et al.*, 2016 ▸). The bright-red spots appearing near atoms O1, O2 and hydrogen atoms H10*A*, H10*B*, H15*A* and H15*B* indicate their roles as the respective donors and/or acceptors; they also appear as blue and red regions corresponding to positive and negative potentials on the HS mapped over electrostatic potential (Spackman *et al.*, 2008 ▸; Jayatilaka *et al.*, 2005 ▸) as shown in Fig. 5 ▸. The blue regions indicate the positive electrostatic potential (hydrogen-bond donors), while the red regions indicate the negative electrostatic potential (hydrogen-bond acceptors). The shape-index of the HS is a tool to visualize π–π stacking by the presence of adjacent red and blue triangles; if there are no adjacent red and/or blue triangles, then there are no π–π inter­actions. Fig. 6 ▸ clearly suggest that there are π–π inter­actions in (I).

**Figure 7:**
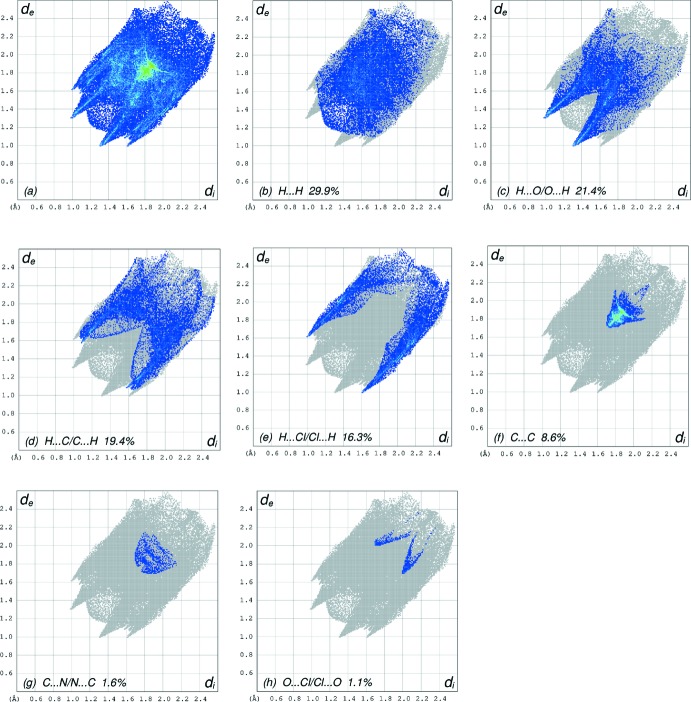
The full two-dimensional fingerprint plots for the title compound, showing (*a*) all inter­actions, and delineated into (*b*) H⋯H, (*c*) H⋯O/O⋯H, (*d*) H⋯C/C⋯H, (*e*) H⋯Cl/Cl⋯H, (*f*) C⋯C, (*g*) C⋯N/N⋯C and (*h*) O⋯Cl/Cl⋯O inter­actions. The d_i_ and d_e_ values are the closest inter­nal and external distances (in Å) from given points on the Hirshfeld surface contacts.

The overall two-dimensional fingerprint plot, Fig. 7 ▸ *a*, and those delineated into H ⋯ H, H⋯O/O⋯H, H⋯C/C⋯H, H⋯Cl/Cl⋯H, C⋯C, C⋯N/N ⋯ C and O⋯Cl/Cl⋯O contacts (McKinnon *et al.*, 2007 ▸) are illustrated in Fig. 7 ▸ *b*–*h*, respectively, together with their relative contributions to the Hirshfeld surface. The most important inter­action is H⋯H (Table 2 ▸), contributing 29.9% to the overall crystal packing, which is reflected in Fig. 7 ▸ *b* as widely scattered points of high density due to the large hydrogen content of the mol­ecule with the tip at *d*_e_ = *d*_i_ = 1.22 Å. The pair of characteristic wings in the fingerprint plot delineated into H⋯O/O⋯H contacts (21.4% contribution, Fig. 7 ▸ *c*) are viewed as a pair of spikes with the tips at *d*_e_ + *d*_i_ = 2.28 Å. In the absence of C—H⋯π inter­actions, the pairs of characteristic wings in Fig. 7 ▸ *d* arise from H⋯C/C⋯H contacts (19.4%) and are viewed as pairs of spikes with the tips at *d*_e_ + *d*_i_ = 2.65 Å and 2.70 Å for the thin and thick spikes, respectively. The scattered points in the pair of wings in the fingerprint plot delineated into H⋯Cl/Cl⋯H (16.3% contribution, Fig. 7 ▸ *e*) have a symmetrical distribution with the edges at *d*_e_ + *d*_i_ = 2.60 Å. The C⋯C contacts, Fig. 7 ▸ *f*, have an arrow-shaped distribution of points with the tip at *d*_e_ = *d*_i_ = 1.72 Å. Finally, the characteristic tip and wings in the fingerprint plots delineated into C⋯N/N⋯C and O⋯Cl/Cl⋯O contacts (1.6% and 1.1% contributions, respectively, Fig. 7 ▸ *g* and 7*h*) have the tips at *d*_e_ = *d*_i_ = 1.73 and 3.70 Å, respectively.

**Figure 8:**
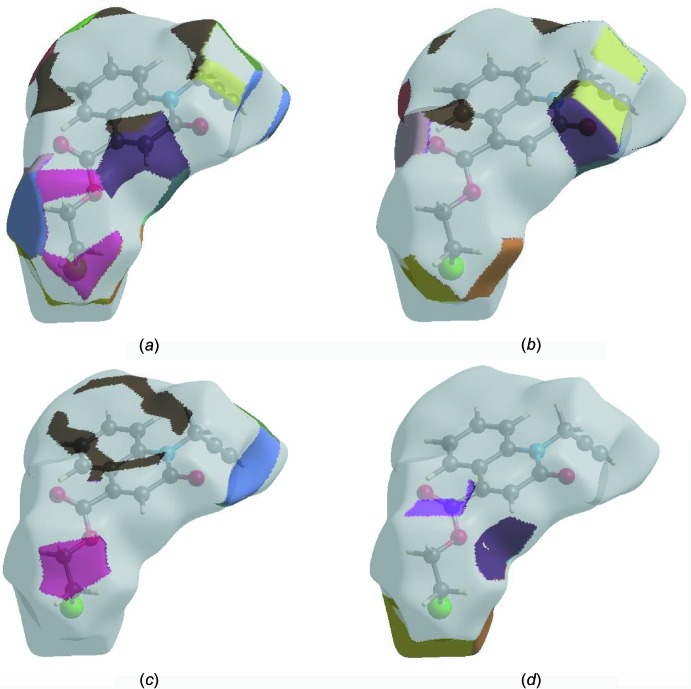
The Hirshfeld surface representations with the function *d*_norm_ plotted onto the surface for (*a*) H⋯H, (*b*) H⋯O/O⋯H, (*c*) H⋯C/C⋯H and (*d*) H⋯Cl/Cl⋯H inter­actions.

The Hirshfeld surface representations with the function *d*_norm_ plotted onto the surface are shown for the H⋯H, H⋯O/O⋯H, H⋯C/C⋯H and H ⋯ Cl/Cl⋯H inter­actions in Fig. 8 ▸ *a*–*d*, respectively.

The Hirshfeld surface analysis confirms the importance of H-atom contacts in establishing the packing. The large number of H⋯H, H⋯O/O⋯H, H ⋯ C/C⋯H and H⋯Cl/Cl⋯H inter­actions suggest that van der Waals inter­actions and hydrogen bonding play the major roles in the crystal packing (Hathwar *et al.*, 2015 ▸).

## Inter­action energy calculations   

The inter­molecular inter­action energies were calculated using the CE–B3LYP/6–31G(d,p) energy model available in *CrystalExplorer17.5* (Turner *et al.*, 2017 ▸), where by default a cluster of mol­ecules are generated by applying crystallographic symmetry operations with respect to a selected central mol­ecule within a radius of 3.8 Å (Turner *et al.*, 2014 ▸). The total inter­molecular energy (*E*_tot_) is the sum of electrostatic (*E*_ele_), polarization (*E*_pol_), dispersion (*E*_dis_) and exchange-repulsion (*E*_rep_) energies (Turner *et al.*, 2015 ▸) with scale factors of 1.057, 0.740, 0.871 and 0.618, respectively (Mackenzie *et al.*, 2017 ▸). Hydrogen-bonding inter­action energies (in kJ mol^−1^) were calculated to be −25.2 (*E*_ele_), −2.1 (*E*_pol_), −85.4 (*E*_dis_), 57.5 (*E*_rep_) and −67.1 (*E*_tot_) for the C—H_Prpn­yl_⋯O_Carbx_ hydrogen bond and −26.5 (*E*_ele_), −4.7 (*E*_pol_), −73.2 (*E*_dis_), 54.3 (*E*_rep_) and −61.7 (*E*_tot_) for the C—H_Chlethy_⋯O_Carbx_ hydrogen bond.

## DFT calculations   

**Figure 9:**
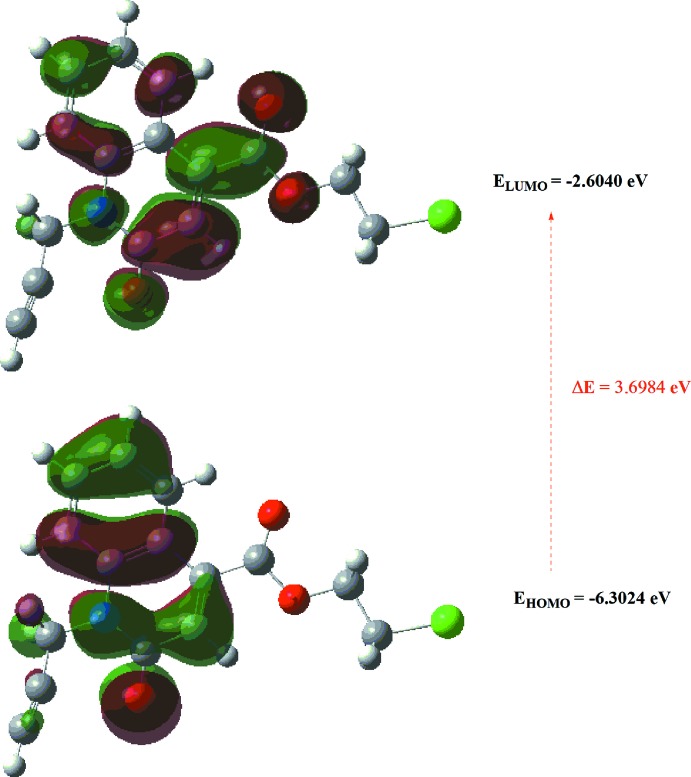
The energy band gap of the title compound.

The optimized structure of the title compound in the gas phase was generated theoretically *via* density functional theory (DFT) using the standard B3LYP functional and 6–311 G(d,p) basis-set calculations (Becke, 1993 ▸) as implemented in *GAUSSIAN 09* (Frisch *et al.*, 2009 ▸). The theoretical and experimental results were in good agreement (Table 3 ▸). The highest-occupied mol­ecular orbital (HOMO), acting as an electron donor, and the lowest-unoccupied mol­ecular orbital (LUMO), acting as an electron acceptor, are very important parameters for quantum chemistry. When the energy gap is small, the mol­ecule is highly polarizable and has high chemical reactivity. The DFT calculations provide some important information on the reactivity and site selectivity of the mol­ecular framework. *E*_HOMO_ and *E*_LUMO_ clarify the inevitable charge-exchange collaboration inside the studied material, and are recorded in Table 4 ▸ along with the electronegativity (χ), hardness (η), potential (μ), electrophilicity (ω) and softness (*σ)*. The significance of η and *σ* is to evaluate both the reactivity and stability. The electron transition from the HOMO to the LUMO energy level is shown in Fig. 9 ▸. The HOMO and LUMO are localized in the plane extending from the whole 2-chloro­ethyl 2-oxo-1-(prop-2-yn-1-yl)-1,2-di­hydro­quinoline-4-carboxyl­ate ring. The energy band gap [Δ*E* = *E*_LUMO_ − *E*_HOMO_] of the mol­ecule is 3.6984 eV, and the frontier mol­ecular orbital energies, *E*_HOMO_ and *E*_LUMO_ are −6.3024 and −2.6040 eV, respectively.

## Database survey   

A non-alkyl­ated analogue, namely quinoline and its derivatives, has been reported (Filali Baba *et al.*, 2016 ▸, 2017 ▸), as well as three similar structures, see: Bouzian *et al.*, 2018 ▸, 2019*a* ▸,*b* ▸; Filali Baba *et al.*, 2019 ▸.

## Synthesis and crystallization   

To a solution of 2-chloro­ethyl 2-oxo-1,2-di­hydro­quinoline-4-carboxyl­ate (0.50 g, 2.00 mmol) in DMF (10.00 ml) were added propargyl bromide (0.20 ml, 2.38 mmol), K_2_CO_3_ (0.82 g, 6.00 mmol) and TBAB (0.06 g, 0.20 mmol). The reaction mixture was stirred at room temperature for 6 h. After removal of the salts by filtration, the solvent was evaporated under reduced pressure and the resulting residue was dissolved in di­chloro­methane. The organic phase was dried with Na_2_SO_4_, and then concentrated under reduced pressure. The pure compound was obtained by column chromatography using hexa­ne/ethyl acetate (3/1) as eluent. The isolated solid was recrystallized from hexa­ne/ethyl acetate (3:1) to afford colourless crystals (yield: 84%, m.p. 394.15 K).

## Refinement   

Crystal data, data collection and structure refinement details are summarized in Table 5 ▸. Hydrogen atoms were positioned geometrically (C—H = 0.95 and 0.99 Å, for CH and CH_2_ H atoms, respectively) and constrained to ride on their parent atoms, with *U*_iso_(H) = 1.2*U*_eq_(C). The largest peak and hole in the final difference map are +0.73 e Å^−3^ (1.00 Å away from Cl1) and −0.35 e Å^−3^ (0.64 Å away from C14), and are associated with the 2-chloro­ethyl­carb­oxy group and may indicate a slight degree of disorder here but it was not considered serious enough to model.

## Supplementary Material

Crystal structure: contains datablock(s) I, global. DOI: 10.1107/S2056989019012283/lh5918sup1.cif

Structure factors: contains datablock(s) I. DOI: 10.1107/S2056989019012283/lh5918Isup2.hkl

Supporting information file. DOI: 10.1107/S2056989019012283/lh5918Isup3.cdx

Supporting information file. DOI: 10.1107/S2056989019012283/lh5918Isup4.cml

CCDC reference: 1951439

Additional supporting information:  crystallographic information; 3D view; checkCIF report

## Figures and Tables

**Table 1:** Hydrogen-bond geometry (Å, °)

*D*—H⋯*A*	*D*—H	H⋯*A*	*D*⋯*A*	*D*—H⋯*A*
C10—H10*A*⋯O2^viii^	0.99	2.49	3.458 (5)	167
C10—H10*B*⋯O1^iv^	0.99	2.39	3.250 (4)	145
C15—H15*A*⋯O1^iii^	0.99	2.46	3.406 (6)	159
C15—H15*B*⋯O2^xi^	0.99	2.40	3.219 (6)	140

Symmetry codes: (iii) 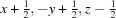; (iv) 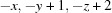; (viii) 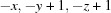; (xi) 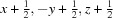.

**Table 2:** Selected interatomic distances (Å)

Cl1⋯O3	3.110 (3)	C1⋯C6^viii^	3.534 (5)
Cl1⋯C12^i^	3.629 (5)	C2⋯C6^ii^	3.489 (5)
Cl1⋯H12^i^	2.75	C2⋯C10^viii^	3.388 (5)
Cl1⋯H5^ii^	3.03	C4⋯C7^viii^	3.597 (5)
Cl1⋯H8^iii^	2.96	C4⋯C9^ii^	3.452 (5)
O1⋯C10^iv^	3.250 (5)	C5⋯C11	3.241 (5)
O1⋯C12^v^	3.409 (6)	C5⋯C9^viii^	3.575 (5)
O1⋯C15^vi^	3.406 (5)	C6⋯C6^viii^	3.485 (4)
O2⋯C2	3.045 (5)	C2⋯H10*A* ^viii^	2.88
O2⋯C15^vii^	3.219 (6)	C5⋯H10*A*	2.61
O3⋯Cl1	3.110 (3)	C10⋯H5	2.50
O1⋯H10*B*	2.30	C11⋯H3^ix^	2.85
O1⋯H10*B* ^iv^	2.39	C11⋯H5	2.72
O1⋯H15*A* ^vi^	2.46	C12⋯H14*A* ^x^	2.95
O2⋯H14*B*	2.46	C12⋯H2^ii^	2.80
O2⋯H2	2.49	C12⋯H3^ix^	2.93
O2⋯H14*A*	2.80	C13⋯H2	2.65
O2⋯H15*B* ^vii^	2.40	H5⋯H10*A*	2.10
O2⋯H10*A* ^viii^	2.49	H8⋯H15*A* ^vi^	2.55
O3⋯H8	2.50		

Symmetry codes: (i) 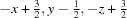; (ii) 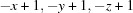; (iii) 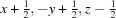; (iv) 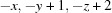; (v) 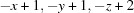; (vi) 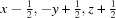; (vii) 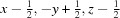; (viii) 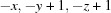; (ix) 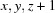; (x) 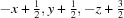.

**Table 3:** Comparison of selected (X-ray and DFT) geometric data (Å, °)

Bonds/angles	X-ray	B3LYP/6–311G(d,p)
Cl1—C15	1.838 (6)	1.88121
O1—C7	1.235 (5)	1.25852
O2—C13	1.213 (5)	1.24099
O3—C13	1.322 (5)	1.38771
O3—C14	1.459 (5)	1.47976
N1—C7	1.381 (5)	1.40545
N1—C6	1.405 (4)	1.41686
N1—C10	1.469 (4)	1.49984
C13—O3—C14	115.2 (4)	116.83182
C7—N1—C6	123.1 (3)	121.89630
C7—N1—C10	116.9 (3)	117.96161
C6—N1—C10	120.0 (3)	117.10486
N1—C6—C1	119.5 (3)	120.53011
O1—C7—N1	121.4 (3)	122.42582
O1—C7—C8	122.5 (3)	121.61064
N1—C7—C8	116.1 (3)	115.96268

**Table 4:** Calculated energies

Mol­ecular Energy	
Total Energy, *TE*	−35893.2971
E_HOMO_ (eV)	−6.3024
E_LUMO_ (eV)	−2.6040
Gap *ΔE* (eV)	3.6984
Dipole moment, *μ* (Debye)	3.8441
Ionization potential, *I* (eV)	6.3024
Electron affinity, *A*	2.6040
Electro negativity, *χ*	4.4532
Hardness, *η*	1.8492
Electrophilicity index, *ω*	5.3620
Softness, *σ*	0.5408
Fraction of electron transferred, *ΔN*	0.6886

**Table 5:** Experimental details

Crystal data	
Chemical formula	C_15_H_12_ClNO_3_
*M*_r_	289.71
Crystal system, space group	Monoclinic, *P*2_1_/*n*
Temperature (K)	150
*a*, *b*, *c* (Å)	7.1809 (2), 21.4466 (5), 8.9173 (2)
β (°)	92.784 (2)
*V* (Å^3^)	1371.70 (6)
*Z*	4
Radiation type	Cu *K*α
μ (mm^−1^)	2.53
Crystal size (mm)	0.19 × 0.14 × 0.01

Data collection	
Diffractometer	Bruker D8 VENTURE PHOTON 100 CMOS
Absorption correction	Multi-scan (*SADABS*; Krause *et al.*, 2015 ▸)
*T*_min_, *T*_max_	0.64, 0.97
No. of measured, independent and observed [*I* > 2σ(*I*)] reflections	10119, 2555, 2170
*R*_int_	0.047
(sin θ/λ)_max_ (Å^−1^)	0.610

Refinement	
*R*[*F* ^2^ > 2σ(*F* ^2^)], *wR*(*F* ^2^), *S*	0.078, 0.178, 1.13
No. of reflections	2555
No. of parameters	181
H-atom treatment	H-atom parameters constrained
Δρ_max_, Δρ_min_ (e Å^−3^)	0.73, −0.35

Computer programs: *APEX3* and *SAINT* (Bruker, 2016 ▸), *SHELXT* (Sheldrick, 2015*a* ▸), *SHELXL2018* (Sheldrick, 2015*b* ▸), *DIAMOND* (Brandenburg & Putz, 2012 ▸) and *SHELXTL* (Sheldrick, 2008 ▸).

## supplementary crystallographic information

### Crystal data

**Table:**

C_15_H_12_ClNO_3_	*F*(000) = 600
*M**_r_* = 289.71	*D*_x_ = 1.403 Mg m^−^^3^
Monoclinic, *P*2_1_/*n*	Cu *K*α radiation, λ = 1.54178 Å
*a* = 7.1809 (2) Å	Cell parameters from 6719 reflections
*b* = 21.4466 (5) Å	θ = 4.1–69.9°
*c* = 8.9173 (2) Å	µ = 2.53 mm^−^^1^
β = 92.784 (2)°	*T* = 150 K
*V* = 1371.70 (6) Å^3^	Plate, colourless
*Z* = 4	0.19 × 0.14 × 0.01 mm

### Data collection

**Table:**

Bruker D8 VENTURE PHOTON 100 CMOS diffractometer	2555 independent reflections
Radiation source: INCOATEC IµS micro–focus source	2170 reflections with *I* > 2σ(*I*)
Mirror monochromator	*R*_int_ = 0.047
Detector resolution: 10.4167 pixels mm^-1^	θ_max_ = 70.1°, θ_min_ = 4.1°
ω scans	*h* = −8→8
Absorption correction: multi-scan (*SADABS*; Krause *et al.*, 2015)	*k* = −26→25
*T*_min_ = 0.64, *T*_max_ = 0.97	*l* = −10→10
10119 measured reflections	

### Refinement

**Table:**

Refinement on *F*^2^	Primary atom site location: dual space
Least-squares matrix: full	Secondary atom site location: difference Fourier map
*R*[*F*^2^ > 2σ(*F*^2^)] = 0.078	Hydrogen site location: inferred from neighbouring sites
*wR*(*F*^2^) = 0.178	H-atom parameters constrained
*S* = 1.13	*w* = 1/[σ^2^(*F*_o_^2^) + (0.0332*P*)^2^ + 4.0657*P*] where *P* = (*F*_o_^2^ + 2*F*_c_^2^)/3
2555 reflections	(Δ/σ)_max_ < 0.001
181 parameters	Δρ_max_ = 0.73 e Å^−^^3^
0 restraints	Δρ_min_ = −0.35 e Å^−^^3^

### Special details

**Table:**

Geometry. All esds (except the esd in the dihedral angle between two l.s. planes) are estimated using the full covariance matrix. The cell esds are taken into account individually in the estimation of esds in distances, angles and torsion angles; correlations between esds in cell parameters are only used when they are defined by crystal symmetry. An approximate (isotropic) treatment of cell esds is used for estimating esds involving l.s. planes.
Refinement. Refinement of F^2^ against ALL reflections. The weighted R-factor wR and goodness of fit S are based on F^2^, conventional R-factors R are based on F, with F set to zero for negative F^2^. The threshold expression of F^2^ > 2sigma(F^2^) is used only for calculating R-factors(gt) etc. and is not relevant to the choice of reflections for refinement. R-factors based on F^2^ are statistically about twice as large as those based on F, and R- factors based on ALL data will be even larger. H-atoms attached to carbon were placed in calculated positions (C—H = 0.95 - 0.99 Å) and included as riding contributions with isotropic displacement parameters 1.2 - 1.5 times those of the attached atoms. The largest peaks and holes in the final difference map are < +/-1 e^-^-/%A^-3^ and are associated with the 2-chloroethylcarboxy group and may indicate a slight degree of disorder here but it was not considered serious enough to model.

### Fractional atomic coordinates and isotropic or equivalent isotropic displacement parameters (Å^2^)

**Table:**

	*x*	*y*	*z*	*U*_iso_*/*U*_eq_	
Cl1	0.7800 (2)	0.24965 (6)	0.45136 (18)	0.0683 (4)	
O1	0.1693 (4)	0.43876 (13)	0.9233 (3)	0.0390 (7)	
O2	0.1917 (5)	0.33835 (15)	0.3272 (4)	0.0569 (9)	
O3	0.3893 (5)	0.30409 (14)	0.5116 (4)	0.0505 (8)	
N1	0.1864 (4)	0.50421 (13)	0.7226 (3)	0.0269 (6)	
C1	0.2615 (5)	0.46384 (17)	0.4782 (4)	0.0282 (8)	
C2	0.2997 (5)	0.47567 (19)	0.3269 (4)	0.0345 (9)	
H2	0.324683	0.441692	0.262595	0.041*	
C3	0.3014 (5)	0.5349 (2)	0.2715 (4)	0.0372 (9)	
H3	0.326460	0.541915	0.169253	0.045*	
C4	0.2661 (5)	0.58513 (19)	0.3654 (4)	0.0363 (9)	
H4	0.267501	0.626395	0.326807	0.044*	
C5	0.2290 (5)	0.57527 (18)	0.5145 (4)	0.0312 (8)	
H5	0.205814	0.609762	0.577824	0.037*	
C6	0.2256 (5)	0.51487 (17)	0.5719 (4)	0.0266 (7)	
C7	0.1967 (5)	0.44600 (17)	0.7888 (4)	0.0296 (8)	
C8	0.2365 (5)	0.39456 (17)	0.6907 (4)	0.0326 (8)	
H8	0.244434	0.353690	0.731581	0.039*	
C9	0.2627 (5)	0.40246 (17)	0.5429 (4)	0.0308 (8)	
C10	0.1343 (5)	0.55651 (17)	0.8183 (4)	0.0295 (8)	
H10A	0.047696	0.584337	0.760268	0.035*	
H10B	0.067963	0.540162	0.904796	0.035*	
C11	0.2966 (6)	0.59261 (18)	0.8741 (4)	0.0346 (9)	
C12	0.4275 (7)	0.6208 (2)	0.9178 (5)	0.0485 (11)	
H12	0.533984	0.643689	0.953389	0.058*	
C13	0.2778 (6)	0.34610 (18)	0.4461 (5)	0.0385 (9)	
C14	0.4018 (8)	0.2450 (2)	0.4316 (6)	0.0595 (14)	
H14A	0.286502	0.220384	0.441323	0.071*	
H14B	0.419443	0.252540	0.323617	0.071*	
C15	0.5603 (9)	0.2122 (2)	0.4990 (6)	0.0629 (14)	
H15A	0.559154	0.168527	0.463120	0.076*	
H15B	0.551442	0.211642	0.609416	0.076*	

### Atomic displacement parameters (Å^2^)

**Table:**

	*U*^11^	*U*^22^	*U*^33^	*U*^12^	*U*^13^	*U*^23^
Cl1	0.0708 (9)	0.0393 (6)	0.0940 (11)	0.0187 (6)	−0.0052 (7)	−0.0096 (6)
O1	0.0464 (16)	0.0416 (15)	0.0301 (15)	0.0056 (13)	0.0132 (12)	0.0061 (12)
O2	0.074 (2)	0.0488 (19)	0.0469 (19)	0.0006 (17)	−0.0037 (17)	−0.0143 (15)
O3	0.057 (2)	0.0357 (16)	0.060 (2)	0.0087 (14)	0.0107 (16)	−0.0126 (14)
N1	0.0262 (15)	0.0270 (15)	0.0282 (16)	0.0024 (12)	0.0081 (12)	−0.0009 (12)
C1	0.0206 (16)	0.0332 (19)	0.0314 (19)	−0.0018 (14)	0.0064 (14)	−0.0014 (15)
C2	0.0276 (19)	0.047 (2)	0.030 (2)	−0.0039 (17)	0.0062 (15)	−0.0071 (17)
C3	0.033 (2)	0.051 (2)	0.028 (2)	−0.0076 (18)	0.0037 (16)	0.0055 (17)
C4	0.033 (2)	0.041 (2)	0.035 (2)	−0.0045 (17)	−0.0001 (16)	0.0091 (17)
C5	0.0264 (18)	0.0325 (19)	0.035 (2)	−0.0008 (15)	0.0035 (15)	0.0018 (16)
C6	0.0194 (16)	0.0335 (19)	0.0273 (18)	0.0005 (14)	0.0058 (13)	0.0004 (15)
C7	0.0252 (18)	0.0302 (19)	0.034 (2)	0.0011 (14)	0.0082 (15)	0.0029 (15)
C8	0.0317 (19)	0.0285 (19)	0.038 (2)	0.0020 (15)	0.0080 (16)	0.0044 (16)
C9	0.0249 (18)	0.0323 (19)	0.036 (2)	0.0006 (14)	0.0088 (15)	−0.0020 (16)
C10	0.0287 (18)	0.0307 (19)	0.0297 (19)	0.0034 (15)	0.0076 (15)	−0.0025 (15)
C11	0.043 (2)	0.034 (2)	0.028 (2)	0.0003 (17)	0.0101 (17)	−0.0039 (16)
C12	0.047 (3)	0.056 (3)	0.043 (3)	−0.009 (2)	0.006 (2)	−0.010 (2)
C13	0.038 (2)	0.030 (2)	0.049 (3)	0.0004 (17)	0.0092 (19)	−0.0001 (18)
C14	0.086 (4)	0.029 (2)	0.065 (3)	0.012 (2)	0.021 (3)	−0.005 (2)
C15	0.091 (4)	0.046 (3)	0.051 (3)	0.013 (3)	0.007 (3)	0.002 (2)

### Geometric parameters (Å, º)

**Table:**

Cl1—C15	1.838 (6)	C5—C6	1.393 (5)
O1—C7	1.235 (5)	C5—H5	0.9500
O2—C13	1.213 (5)	C7—C8	1.445 (5)
O3—C13	1.322 (5)	C8—C9	1.351 (5)
O3—C14	1.459 (5)	C8—H8	0.9500
N1—C7	1.381 (5)	C9—C13	1.492 (5)
N1—C6	1.405 (4)	C10—C11	1.465 (5)
N1—C10	1.469 (4)	C10—H10A	0.9900
C1—C6	1.409 (5)	C10—H10B	0.9900
C1—C2	1.412 (5)	C11—C12	1.169 (6)
C1—C9	1.437 (5)	C12—H12	0.9500
C2—C3	1.363 (6)	C14—C15	1.444 (8)
C2—H2	0.9500	C14—H14A	0.9900
C3—C4	1.396 (6)	C14—H14B	0.9900
C3—H3	0.9500	C15—H15A	0.9900
C4—C5	1.385 (5)	C15—H15B	0.9900
C4—H4	0.9500		
			
Cl1···O3	3.110 (3)	C1···C6^viii^	3.534 (5)
Cl1···C12^i^	3.629 (5)	C2···C6^ii^	3.489 (5)
Cl1···H12^i^	2.75	C2···C10^viii^	3.388 (5)
Cl1···H5^ii^	3.03	C4···C7^viii^	3.597 (5)
Cl1···H8^iii^	2.96	C4···C9^ii^	3.452 (5)
O1···C10^iv^	3.250 (5)	C5···C11	3.241 (5)
O1···C12^v^	3.409 (6)	C5···C9^viii^	3.575 (5)
O1···C15^vi^	3.406 (5)	C6···C6^viii^	3.485 (4)
O2···C2	3.045 (5)	C2···H10A^viii^	2.88
O2···C15^vii^	3.219 (6)	C5···H10A	2.61
O3···Cl1	3.110 (3)	C10···H5	2.50
O1···H10B	2.30	C11···H3^ix^	2.85
O1···H10B^iv^	2.39	C11···H5	2.72
O1···H15A^vi^	2.46	C12···H14A^x^	2.95
O2···H14B	2.46	C12···H2^ii^	2.80
O2···H2	2.49	C12···H3^ix^	2.93
O2···H14A	2.80	C13···H2	2.65
O2···H15B^vii^	2.40	H5···H10A	2.10
O2···H10A^viii^	2.49	H8···H15A^vi^	2.55
O3···H8	2.50		
			
C13—O3—C14	115.2 (4)	C7—C8—H8	118.8
C7—N1—C6	123.1 (3)	C8—C9—C1	120.5 (3)
C7—N1—C10	116.9 (3)	C8—C9—C13	118.7 (3)
C6—N1—C10	120.0 (3)	C1—C9—C13	120.6 (3)
C6—C1—C2	118.5 (3)	C11—C10—N1	112.3 (3)
C6—C1—C9	118.1 (3)	C11—C10—H10A	109.1
C2—C1—C9	123.4 (3)	N1—C10—H10A	109.1
C3—C2—C1	121.3 (4)	C11—C10—H10B	109.1
C3—C2—H2	119.4	N1—C10—H10B	109.1
C1—C2—H2	119.4	H10A—C10—H10B	107.9
C2—C3—C4	119.8 (4)	C12—C11—C10	179.1 (5)
C2—C3—H3	120.1	C11—C12—H12	180.0
C4—C3—H3	120.1	O2—C13—O3	124.4 (4)
C5—C4—C3	120.5 (4)	O2—C13—C9	124.6 (4)
C5—C4—H4	119.8	O3—C13—C9	110.8 (4)
C3—C4—H4	119.8	C15—C14—O3	106.6 (5)
C4—C5—C6	120.1 (4)	C15—C14—H14A	110.4
C4—C5—H5	119.9	O3—C14—H14A	110.4
C6—C5—H5	119.9	C15—C14—H14B	110.4
C5—C6—N1	120.7 (3)	O3—C14—H14B	110.4
C5—C6—C1	119.8 (3)	H14A—C14—H14B	108.6
N1—C6—C1	119.5 (3)	C14—C15—Cl1	111.0 (4)
O1—C7—N1	121.4 (3)	C14—C15—H15A	109.4
O1—C7—C8	122.5 (3)	Cl1—C15—H15A	109.4
N1—C7—C8	116.1 (3)	C14—C15—H15B	109.4
C9—C8—C7	122.4 (3)	Cl1—C15—H15B	109.4
C9—C8—H8	118.8	H15A—C15—H15B	108.0
			
C6—C1—C2—C3	−0.3 (5)	O1—C7—C8—C9	178.7 (4)
C9—C1—C2—C3	−178.4 (4)	N1—C7—C8—C9	0.1 (5)
C1—C2—C3—C4	0.4 (6)	C7—C8—C9—C1	3.3 (6)
C2—C3—C4—C5	−0.1 (6)	C7—C8—C9—C13	−171.9 (3)
C3—C4—C5—C6	−0.4 (6)	C6—C1—C9—C8	−2.3 (5)
C4—C5—C6—N1	−179.4 (3)	C2—C1—C9—C8	175.9 (4)
C4—C5—C6—C1	0.5 (5)	C6—C1—C9—C13	172.9 (3)
C7—N1—C6—C5	−174.3 (3)	C2—C1—C9—C13	−9.0 (5)
C10—N1—C6—C5	4.7 (5)	C7—N1—C10—C11	99.5 (4)
C7—N1—C6—C1	5.7 (5)	C6—N1—C10—C11	−79.6 (4)
C10—N1—C6—C1	−175.2 (3)	C14—O3—C13—O2	−0.9 (6)
C2—C1—C6—C5	−0.2 (5)	C14—O3—C13—C9	175.3 (4)
C9—C1—C6—C5	178.0 (3)	C8—C9—C13—O2	131.0 (5)
C2—C1—C6—N1	179.7 (3)	C1—C9—C13—O2	−44.2 (6)
C9—C1—C6—N1	−2.1 (5)	C8—C9—C13—O3	−45.2 (5)
C6—N1—C7—O1	176.8 (3)	C1—C9—C13—O3	139.6 (4)
C10—N1—C7—O1	−2.3 (5)	C13—O3—C14—C15	166.2 (4)
C6—N1—C7—C8	−4.7 (5)	O3—C14—C15—Cl1	−70.8 (5)
C10—N1—C7—C8	176.2 (3)		

Symmetry codes: (i) −*x*+3/2, *y*−1/2, −*z*+3/2; (ii) −*x*+1, −*y*+1, −*z*+1; (iii) *x*+1/2, −*y*+1/2, *z*−1/2; (iv) −*x*, −*y*+1, −*z*+2; (v) −*x*+1, −*y*+1, −*z*+2; (vi) *x*−1/2, −*y*+1/2, *z*+1/2; (vii) *x*−1/2, −*y*+1/2, *z*−1/2; (viii) −*x*, −*y*+1, −*z*+1; (ix) *x*, *y*, *z*+1; (x) −*x*+1/2, *y*+1/2, −*z*+3/2.

### Hydrogen-bond geometry (Å, º)

**Table:**

*D*—H···*A*	*D*—H	H···*A*	*D*···*A*	*D*—H···*A*
C10—H10*A*···O2^viii^	0.99	2.49	3.458 (5)	167
C10—H10*B*···O1^iv^	0.99	2.39	3.250 (4)	145
C15—H15*A*···O1^iii^	0.99	2.46	3.406 (6)	159
C15—H15*B*···O2^xi^	0.99	2.40	3.219 (6)	140

Symmetry codes: (iii) *x*+1/2, −*y*+1/2, *z*−1/2; (iv) −*x*, −*y*+1, −*z*+2; (viii) −*x*, −*y*+1, −*z*+1; (xi) *x*+1/2, −*y*+1/2, *z*+1/2.
